# What Drives the Diversity of the Most Abundant Terrestrial Cercozoan Family (Rhogostomidae, Cercozoa, Rhizaria)?

**DOI:** 10.3390/microorganisms8081123

**Published:** 2020-07-26

**Authors:** Hüsna Öztoprak, Susanne Walden, Thierry Heger, Michael Bonkowski, Kenneth Dumack

**Affiliations:** 1 Institute of Zoology, Terrestrial Ecology, University of Cologne, Zülpicher Str. 47b, 50674 Köln, Germany; h.oeztoprak@uni-koeln.de (H.Ö.); swalden1@uni-koeln.de (S.W.); m.bonkowski@uni-koeln.de (M.B.); 2 Soil Science and Environment Group, CHANGINS, University of Applied Sciences and Arts Western Switzerland, Route de Duillier 50, 1260 Nyon, Switzerland; thierry.heger@changins.ch

**Keywords:** biogeography, environmental drivers, protists, thecate amoebae, morphological traits, soil

## Abstract

Environmental sequencing surveys of soils and freshwaters revealed high abundance and diversity of the Rhogostomidae, a group of omnivorous thecate amoebae. This is puzzling since only a few Rhogostomidae species have yet been described and only a handful of reports mention them in field surveys. We investigated the putative cryptic diversity of the Rhogostomidae by a critical re-evaluation of published environmental sequencing data and in-depth ecological and morphological trait analyses. The Rhogostomidae exhibit an amazing diversity of genetically distinct clades that occur in a variety of different environments. We further broadly sampled for Rhogostomidae species; based on these isolates, we describe eleven new species and highlight important morphological traits for species delimitation. The most important environmental drivers that shape the Rhogostomidae community were soil moisture, soil pH, and total plant biomass. The length/width ratio of the theca was a morphological trait related to the colonized habitats, but not the shape and size of the aperture that is often linked to moisture adaption in testate and thecate amoebae.

## 1. Introduction

Environmental sequencing surveys challenge traditional beliefs of protist biodiversity and its environmental drivers. Amplicon-based metagenomics (also called metabarcoding) with different underlying bioinformatic pipelines and various primer sets all report consistent findings: (i) Cercozoa (Rhizaria) are one of the most abundant groups of protists in soils [1,2,3] and (ii) among the cercozoan sequence reads, operational taxonomic units (OTUs) assigned to the Rhogostomidae (Thecofilosea) appear to dominate cercozoan communities in a wide variety of terrestrial and some aquatic habitats [4,5,6,7,8,9,10]. However, aside from such molecular surveys, the Rhogostomidae are hardly mentioned in scientific literature and only a handful of species have been described until today [11,12,13]. Consequently, there appears to exist high untapped biodiversity of potentially important microbial key players, which we here investigated comprehensively taking the family Rhogostomidae as an example.

Theodosius Dobzhansky, half a century ago, made an iconic statement, i.e., "nothing in biology makes sense except in the light of evolution” [14]. With decreasing costs, environmental sequencing produces huge amounts of microbial data and the meaningful interpretation of the data becomes the main effort. The correct exploitation of environmental data, including the assignment of genetic data, in this case OTUs, to existing species is crucial for linking microbial sequence diversity to functioning [15]. In other words: Nothing in environmental sequencing makes sense unless it is based on species identity. However, since the biological species concept cannot be applied to most protists, which generally reproduce asexually, it is still a controversial topic what a protist species is and how it could be defined [16,17]. ‘Integrative taxonomy’ appears a promising way forward to overcome the current ‘taxonomy crisis’ by delimiting, identifying and integrating a broad range of characteristics (i.e., comparative morphology, population genetics, phylogeography, and ecology) [18,19]. The concept of integrative taxonomy in protistology, however, is still in its infancy.

Shell-bearing protists have long been in focus of protistologists and ecologists [20]. Shells of amoebae can be (i) tests, which are composed of solid and persistent compounds, such as silica scales, siliceous rods or spines, agglutinated quartz grains or empty diatom frustules, or either entirely built of proteinaceous material or (ii) thecae, which are to a certain degree flexible, transparent, adherent to the cell body, usually colorless and most often free of agglutinated materials. In comparison to testate amoebae, thecate amoebae were long neglected [21,22]. The family Rhogostomidae belongs to the Cryomonadida (Thecofilosea, Cercozoa) and is comprised of three genera: *Capsellina*, *Sacciforma,* and *Rhogstoma*. Prior to this study, only five to six *Rhogostoma* species were described (depending on whether “*Capsellina timida*” is a genuine *Rhogostoma* species). Two *Rhogostoma* species were described during the erection of the genus by Belar in 1921 [23] and three species were added almost a whole century after the genus was established (for an overview of described species and their characters see [11]). Recent investigations, however, indicated that morphological identification and species delimitation is particularly difficult in this taxon since species of the Rhogostomidae are typically very small (<15 µm in diameter) and lack strikingly distinct morphological characters [11,13]. 

The major aim of this explorative study was a general characterization of *Rhogostoma* on a large scale, thus including genetic, morphological, and ecological characterization. Accordingly, the following hypotheses were raised:

1. Rhogostomidae are exceptionally species-rich.
2. Rhogostomidae are ubiquitously distributed, including marine, freshwater, and terrestrial habitats.
3. *Rhogostoma* derived from aquatic relatives and colonized terrestrial habitats.
4. *Rhogostoma* adapted to terrestrial environments with a deeper invagination of the aperture and a less elongated, but more spherical, cell shape to reduce water loss.

In an attempt to uncover the hidden diversity of the Rhogostomidae, we (i) isolated 16 different *Rhogostoma* strains from freshwater and terrestrial habitats, sequenced their SSU rDNA and subjected these, together with already published sequences, to phylogenetic analyses. (ii) We measured morphological traits in these strains, which were subjected to ordination techniques to identify the best suited morphological characters for species delimitation of Rhogostomidae. (iii) We extracted all sequences that could be assigned to the Rhogostomidae from the NCBI database and five publicly available environmental sequencing datasets [5,6,9,24,25] to explore genetic diversity on a large scale. (iv) Finally, we delimit edaphic factors that shape the community composition of Rhogostomidae, based on three of the previously mentioned datasets that come with extensive environmental data [6,24,25]. Based on the consensus of morphological, genetic, and ecological data, we describe eleven novel species grouped into four newly defined Rhogostomidae morphotypes and shed light on the large diversity of Rhogostomidae that yet awaits exploration. 

## 2. Material and Methods

### 2.1. Sampling and Culturing 

Natural samples of soil, freshwater, and marine habitats were screened for Rhogostomidae. Rhogostomidae cells were exclusively found in soil and freshwater samples (Table 1). Isolates were transferred to 24-well plates (SARESTEDT AG & Co. KG, Nümbrecht, Germany) which either contained Waris-H medium [26] for samples containing environmental microalgae, or wheat grass (WG)-medium to facilitate slow growth of environmental bacteria [27]. In all cases, *E. coli* was added as a food source. We were able to obtain 16 cultures of clonal *Rhogostoma* strains. All strains were sub-cultured in culture flasks (SARESTEDT AG & Co. KG, Nümbrecht, Germany) and stored at 14–16 °C. Strains were sub-cultivated monthly and deposited in the Culture Collection for Algae and Protozoa (CCAP). Respective accession numbers are given in Table 1.

### 2.2. Microscopic Observation

Light microscopic observations were made with a Nikon Eclipse TS100 inverted microscope (up to 400× magnification, phase contrast). Pictures and videos were taken with a Nikon Eclipse 90i microscope (DIC, up to 600× magnification) with a Nikon digital sight DS-U2 mounted camera (program: NIS-Elements V4.13.04). Picture assemblies were made with Adobe Photoshop CC 2014 (Adobe Systems, Munich, Germany).

Hapantotypes were dried and submitted to the Upper Austrian State Museum Invertebrate Collection as Inv. Nr. 2019/71-80.

### 2.3. Phylogenetic Analyses

A nearly full-length SSU rDNA sequence was amplified from each isolate in two overlapping fragments, using EukA and EukB as universal eukaryotic primers and different combinations of cercozoan specific primers (Table 1 and Table 2). PCR amplifications were preferably done with single cells in a total volume of 17 µL. The mixture included 1x Thermo Scientific Dream Taq Green Buffer, 1 µM forward and 1 µM reverse primer, 0.2 mM dNTPs, and 0.01× DreamTaq polymerase (Thermo Fisher Scientific, Dreieich, Germany) and _dd_H_2_0 to fill until 17 µL. An amplification profile consisting of 34 cycles with 32 s at 95 °C, 36 s at 50 °C, and 2 min at 72 °C, followed by 7 min at 72 °C for the final extension was conducted. PCR products were purified by adding 1 U/mL of Exonuclease, 0.3 U/mL FastAP and to 8 µL PCR product, then heating for 30 min at 37 °C, and subsequently for 20 min at 85 °C. For sequencing, the Big dye Terminator Cycle sequencing Kit and an ABI PRISM automatic sequencer were used. 

For the phylogenetic analyses, a reference database containing sequences with >93% similarity to the type species *Rhogostoma schuessleri* were obtained from the NCBI GenBank database. These sequences most likely represent the full genetic diversity of Cryomonadida present in the NCBI GenBank database (last date of accession: 26/07/19). Subsequently, mismatches and erroneous sequences were deleted. Eleven sequences of the Tectofilosida were added as outgroup. They were aligned in mafft using the linsi algorithm [30]. The sequences of all strains obtained in this study (Table 1) were manually checked for sequencing errors using Chromas (V2.6.5) and assembled in SeaView (V4.6, [31]). Additionally, we added subsets of large-scale environmental survey data marking the hypervariable region V4 of the SSU rDNA (18S rDNA) of Cryomonadida from following published datasets: Degrune et al. [9], Fiore-Donno et al. [5,6], Heger et al. [25] and Jauss et al. [24]. Accordingly, an alignment with 752 sequences, containing 501 NCBI database sequences and an additional 251 OTUs from the previously mentioned datasets were used for phylogenetic analysis. Maximum likelihood trees were calculated in RAxML (Randomized Axelerated Maximum Likelihood; [32]). The best scoring tree was used to report the confidence values as percentages obtained through 200 non-parametric bootstraps under the GTRCAT model. The same method was used to obtain the 18S rDNA phylogenetic analysis of Rhogostomidae. There, an alignment with all described Rhogostomidae spp. of which SSU rDNA data was publicly available was conducted and eight of the same Tectofilosida spp. as mentioned above were used as outgroup. 

### 2.4. Statistical Analyses

All statistical analyses were carried out in R (V3.6, [33]). To determine significant differences between the morphological features of *Rhogostoma* spp. strains, one-way ANOVA followed by Turkey’s HSD test were conducted. Results were expressed by means and standard deviation.

**Figure 1:**
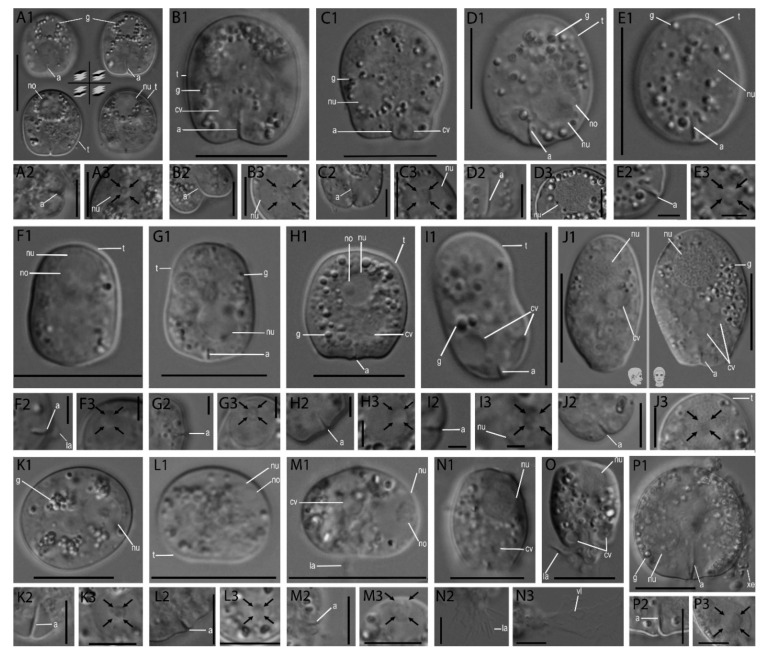
Cellular features of *Rhogostoma* spp. found in this study. Pictures of each strain are divided into (**1**) cell body as an overview (scale bar 10 µm); (**2**) close-up of the aperture (scales bars 5 µm) (**3**) close-up of the nucleus, with the nucleolus being highlighted by arrows (scales bars 5 µm). (**A**): *R. kyoshii* strain WM, (**B**): *R. medica* strain il-I, (**C**): *R. epiphylla* strain IGS, (**D**): *R. karsteni* strain 3A, (**E**): *R. tahiri* strain B10, (**F**): *R. florae* strain K8, (**G**): *R. florae* strain K9, (**H**): *R. leviosa* strain W2, (**I**): *R. pseudocylindrica* strain RC, (**J**): *R. kappa* strain 1A, (**K**): *R. epiphylla* strain WH4, (**L**): *R. absidea* strain W3, (**M**): *R. micra* strain 1B, (**N**): *R. radagasteri* strain TG4-II, (**O**): *R. radagsteri* strain TG-IV, (**P**): *R. bowseri* strain B14. Abbreviations: aperture (a), contractile vacuoles (cv), granules (g), lamellipodia (la), nucleolus (no), nucleus (nu), theca (t), vacuole (vl), xenosomes (xe).

Shifts in the magnitude of morphological traits between isolated Rhogostomidae strains were shown in box plots (see Supplementary Materials). To identify the most significant discriminating morphological traits between strains the traits were normalized by cell size and analyzed by principal component analysis (PCA) using Euclidean distances [34]. Scaling 1 was used to visualize distances among normalized morphological measures in the biplot as approximations of their Euclidean distances in multidimensional space. The radius of the circle of equilibrium contribution represents the length of the vector representing a variable that would contribute equally to all the dimensions of the PCA space (Figure 1; [34]). Non-metric multidimensional scaling (NMDS) using Bray Curtis distances was used to visualize the relationships among beta diversity of Cryomonadida and environmental factors. We used Permutational Multivariate Analysis of Variance (PERMANOVA; [35]) to test if these environmental factors were significantly influencing the Cryomonadida community. Each test was permuted 999 times. All ordinations were calculated with the package vegan [36]. 

## 3. Results

### 3.1. Morphological Analysis 

We were able to culture 16 *Rhogostoma* strains out of approximately 50 screened samples from Germany, the Netherlands, and Austria. All isolated thecate amoebae were bilateral symmetric, bore a smooth hyaline theca, and the cells moved with aid of filopodia which originated from a basal slit-like aperture (Figure 1). Filopodia branched and anastomosed in all directions during the early stages of culturing, but cells in older cultures often ceased to extend their filopodia. According to our analyses, four general *Rhogostoma* morphotypes can be distinguished despite high morphological variability: i.e., cells that were elongated and lateral flattened (*R. kappa*), cylindrical/conical (*R. cylindrica*, *R. pseudocylindrica*, *R. florae*, *R. absidea*, *R. karsteni*), angular (*R. kyoshii*, *R. medica*, *R. radagasteri*) and lateral compressed spherical (*R. schuessleri*, *R. micra*, *R. minus*, *R. epiphylla*, *R. leviosa*, *R. bowseri*). See Supplementary Table S1 for detailed morphological measurements; and Taxonomic Appendix A for detailed species descriptions.

**Figure 2:**
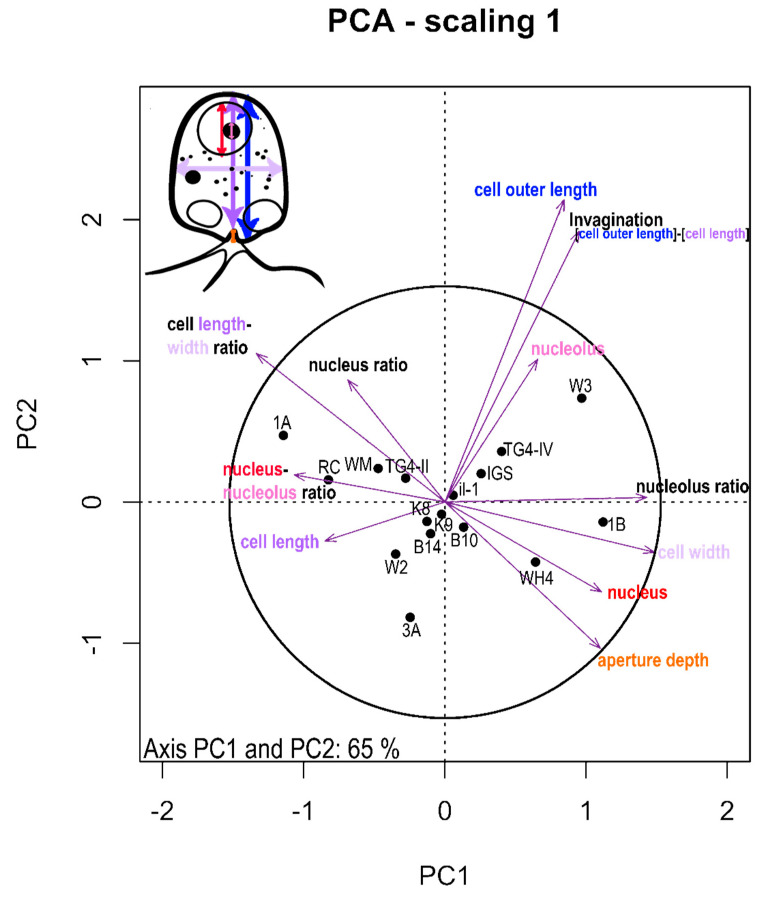
Principal component analysis (PCA)—Scaling 1 = distance plot; the radius of the circle of equilibrium contribution represents the length of the vector of a variable that would contribute equally to all the dimensions of the PCA space. Percentage of the total variability in the scatter plot: axis PC1: 46% and PC2: 19%. Measured morphological traits are depicted in the drawing on the upper left.

To determine important morphological traits for species delimitation, we analyzed traits of *Rhogostoma* spp. via a principal component analysis. Cell length to width ratio, relative cell outer length, and invagination of the aperture turned out to be the morphological traits that were best suited for separating the strains (Figure 2; PCA axis PC1 = 46%; PC2 = 19%). Cell length to width ratio and aperture depth were negatively correlated (*R*^2^ = 0.49, *p* < 0.001). Aperture invagination was positively correlated with cell outer length (*R*^2^ = 0.94, *p* < 0.001). The remaining morphological traits did not contribute sufficiently in explaining variation among strains (see the circle of equilibrium contribution, Figure 2). 

### 3.2. Phylogeny 

**Figure 3:**
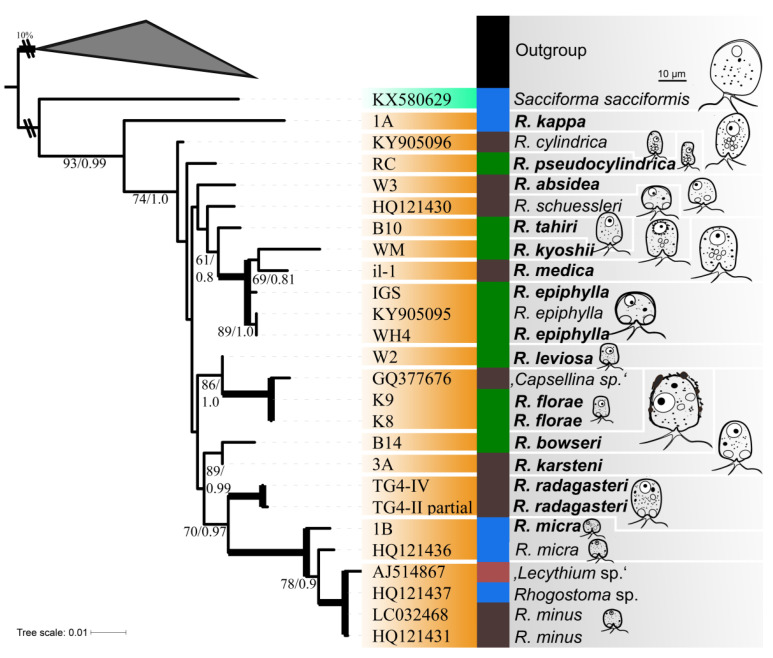
SSU rDNA phylogeny of sequenced and described Rhogostomidae (highlighted in orange for *Rhogostoma* spp. and cyan for *Sacciforma*) with chosen Tectofilosida as outgroup. Shown is the maximum likelihood tree obtained by the RAxML GTR+I+G analyses including 34 sequences. Species names of in this study sequenced strains are highlighted in bold. Support values are given in bootstraps and posterior probabilities (BT/PP) if BT was ≥50%; bold branches indicate bootstrap support of 100%. Isolation habitats are highlighted: blue indicates freshwater origin, brown indicates soil origin, green indicates strains from the phyllosphere and red indicates wastewater treatment plant origin. Drawings of Rhogostomidae, highlighting their morphological traits, are given to scale, enabling a quick visual comparison of each species. Scale bar indicates 10 μm.

To illustrate morphological adaption during the evolution of Rhogostomidae, 16 SSU rDNA sequences were obtained and subjected to phylogenetic analyses. The sequences ranged from 909 to 1767 nucleotides (Table 1). A combined analysis of these sequences and sequences from all currently sequenced and described Rhogostomidae revealed the freshwater genus *Sacciforma* (Rhogostomidae) to group basal to *Rhogostoma* spp. with full support (Figure 3). All *Rhogostoma* spp. group in a highly supported monophylum. Species which originated from freshwater are found in two distinct groups one being basal to (*S. sacciformis* and *R. kappa*) and another one being high in the *Rhogostoma* radiation (*R. radagasteri*, *R. micra* and *R. minus*). The latter intermingles with sequences that originated from soil taxa (Figure 3). Morphological variation did not show a clear pattern with phylogenetic branching, but it must be addressed that some branches within the Rhogostomidae were only poorly supported. 

**Figure 4:**
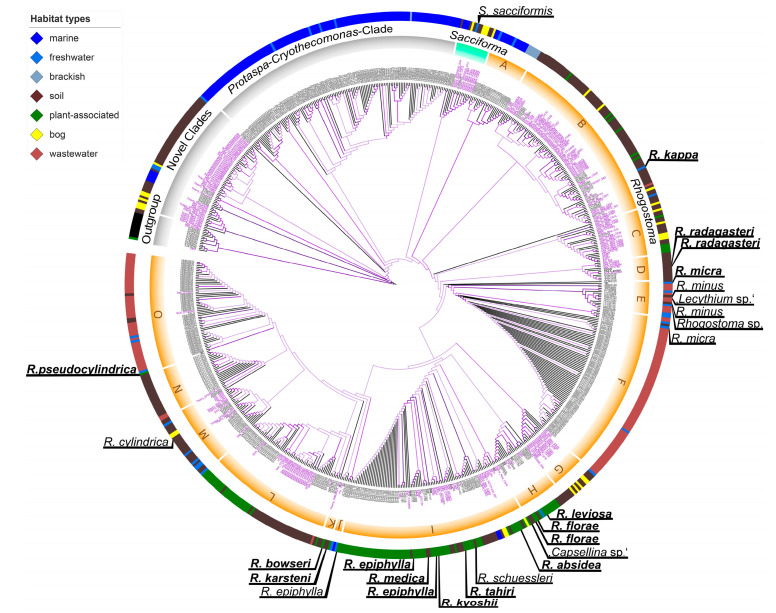
SSU rDNA phylogeny of Cryomonadida (Rhogostomidae and relatives) with a focus on Rhogostomidae diversity (highlighted in orange for *Rhogostoma* and cyan for *Sacciforma*) with chosen Tectofilosida as outgroup. Shown is the maximum likelihood tree obtained by RAxML. The bootstrap values are visualized in a colour flow from black (low support) to purple (high support); end nodes are also given in black. Environmental sequences of the in-depth analysed datasets [5,6,9,24,25] are indicated in purple font, NCBI database environmental sequences and sequences derived from isolated strains are indicated in black. Isolation habitats are highlighted and visualized in the outer colour stripe.

A large-scale phylogenetic analysis was conducted to reveal the genetic diversity of the Rhogostomidae and clades of which strains have not yet been cultured and novel clades that await exploration. Accordingly, we included as many OTUs and their respective metadata as we could gather from published data sets including the NCBI to shed light on the potential distribution of respective clades. The analysis revealed four novel clades at the base of Cryomonadida with sequences originating from diverse environments (Figure 4). Sequences from the genera *Protaspa* and *Cryothecomonas* intermingle with each other and a large variety of environmental OTUs that exclusively originated from saline habitats. Similar to the phylogenetic analyses presented in Figure 3, the genus *Sacciforma* groups basal to *Rhogostoma*. The genus *Rhogostoma* shows a large variety in SSU rDNA sequences and numerous environmental clades without cultured representatives.

#### The Phylogenetic Position of *Capsellina*

The poor quality of the only available sequence of the Rhogostomidae genus *Capsellina* (GQ377676) was already discussed in Dumack et al. [12]. Now we provide further evidence that the name of this sequence is based on misidentification: *Rhogostoma* strains K8 and K9, here described as *R. florae* have a highly similar SSU rDNA sequence to the claimed “*Capsellina* sp.”.

### 3.3. Environmental Drivers of Species Turnover in Rhogostomidae

**Figure 5:**
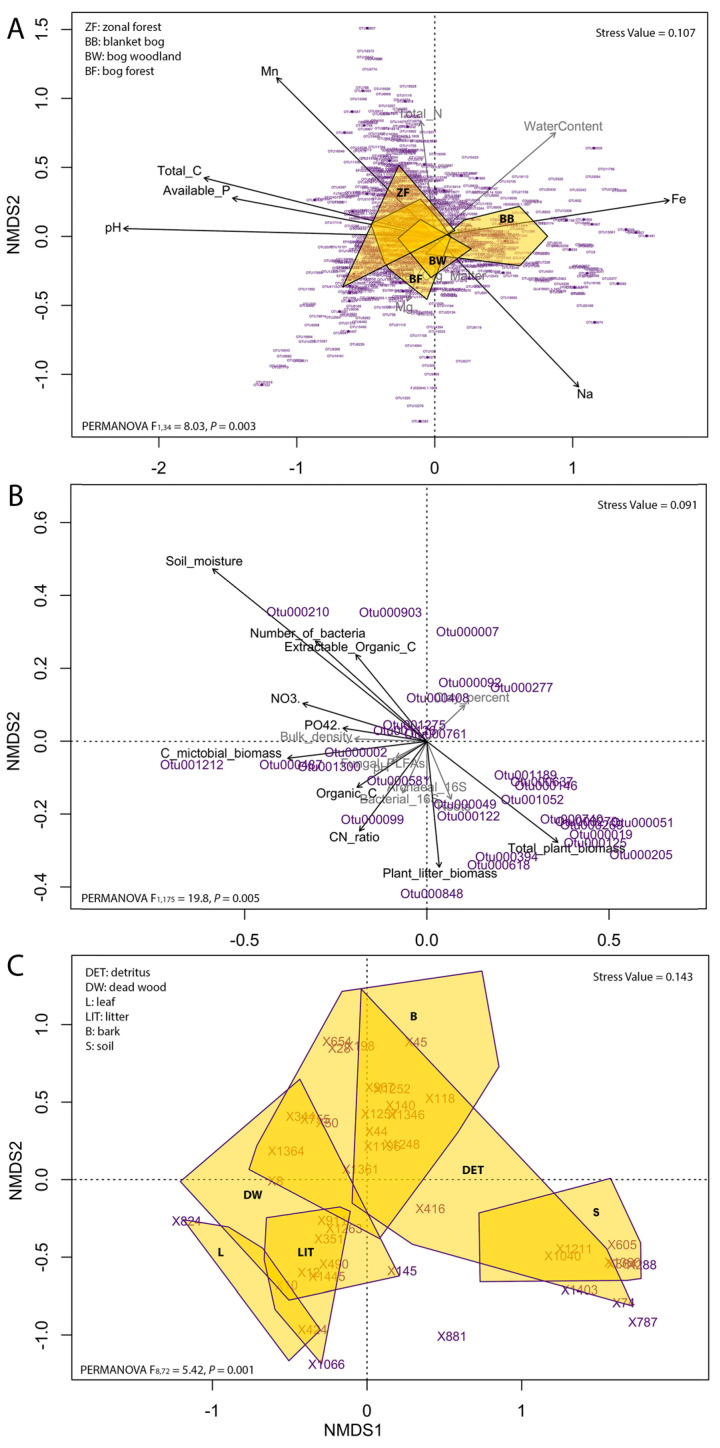
Non-metric multidimensional scaling (NMDS) based on Bray-Curtis distance of relative abundances of environmental parameters. (**A**): Heger et al. [25]; (**B**): Fiore-Donno et al. [7]; (**C**): Jauss et al. [24]. Stress values are shown in the upper right of the graphs. PERMANOVA results are shown in the down left. OTUs are highlighted in purple. Parameters with significant influence on Cryomonadida community are highlighted in bold vectors (**A**,**B**).

Based on previously published datasets that come with extensive metadata [6,24,25], we investigated the effects of soil physicochemical parameters on the beta diversity of Cryomonadida (Rhogostomidae and relatives). We decided to include whole Cryomonadida diversity to avoid exclusion of OTUs with unknown family-affiliation on the basis of an arbitrary threshold. On large scale, the composition of Cryomonadida communities differed among four dominant ecosystem types along a bog-woodland transect (i.e., bog woodland, bog forest, blanket bog, and zonal forest; PERMANOVA F_3,33_ = 4.03, *p* = 0.001). In general, differences in soil physicochemical parameters in respect to soil contents of iron (Fe), sodium (Na), manganese (Mn), total carbon (Total C), available P, and pH influenced species turnover in Cryomonadida (Figure 5A; PERMANOVA F_1,34_ = 8.03, *p* = 0.003). Chemical elements including iron and sodium were among the strongest predictors of Cryomonadida communities, followed closely by soil pH, which ranged in this study from 3.68 to 4.93.

On a small scale, biotic factors like total plant biomass, plant litter biomass, microbial biomass carbon content (Cmic), and numbers of bacteria, as well as soil physicochemical parameters, such as bulk density, soil moisture, soil C/N ratio, organic carbon content (organic C), extractable organic carbon phosphate (PO_4_^2^), and nitrate (NO^3−^) content, had significant effects on the beta diversity of Cryomonadida (Figure 5B; PERMANOVA F_1,175_ = 19.8, *p* = 0.005). Compared to the large-scale bog-woodland transect, soil pH and clay content did not show an effect in the small-scale study in grasslands. Here the strongest predictors of beta diversity of Cryomonadida were soil moisture and its negative correlation with plant biomass (*R*^2^ = 0.28, *p* < 0.001). In a data set comparing protists from tree canopies with those on the forest ground, the Cryomonadida communities differed clearly between canopy habitats and the forest floor, but not among canopy microhabitats such as fresh leaves, bark, deadwood, and detritus between tree branches (Figure 5C; PERMANOVA F_8,72_ = 5.42, *p* = 0.001). 

## 4. Discussion

In this study, we followed an integrative approach to facilitate species delimitation of morphologically quite similar, but genetically distinct minute thecate amoebae. This approach allowed us to identify specific morphological traits and biotic and abiotic environmental drivers to differentiate species in Rhogostomidae and appears as a promising strategy for the reliable species delimitations in cryptic species complexes of protists. We follow the premise that undoubtedly the best taxonomic concept is the one that reflects evolution. At the core of taxonomy lies the notion of the species. Since sex in most protists is decoupled from reproduction, the ‘biological species concept’ cannot be applied [37,38,39]. Following an integrative approach settles the dispute whether morphology or genetic data should be used to delimit species. We show that a combination of both is needed, especially in taxa with few morphological characters, but high genetic diversity, like in Rhogostomidae. Based on the combination of morphological investigations and genetic analyses, we were able to identify reliable morphological traits differentiating species in Rhogostomidae like cell length-to-width ratio and aperture invagination. 

We show that the Cryomonadida are surprisingly diverse and can be isolated from various habitats. We found a high genetic diversity in a single protist genus and almost every isolated individual varied in numerous morphological aspects and thus we conclude the Rhogostomidae to be highly species-rich, confirming our first hypothesis. The consensus of all available environmental sequencing data of the NCBI database and the fact that we were not able to isolate Rhogostomidae from our marine samples indicates a prevalence of Rhogostomidae for terrestrial (soil, plant surfaces) and some freshwater microhabitats (ponds and wastewater treatment plants). According to the wealth of ribosomal sequences in the NCBI database, the Rhogostomidae largely lack representation in marine environments in our data, although marine habitats were in comparison to terrestrial environments highly sampled in recent years [40,41]. According to the branching patterns of the phylogenetic analysis, the Rhogostomidae may have derived from marine ancestors (related to *Protaspa*, *Crypthecomonas*) and subsequently adapted over freshwater (*Sacciforma* and *Rhogostoma kappa*) to terrestrial habitats (most other *Rhogostoma* spp.), leading us to reject our second hypothesis that Rhogostomidae are ubiquitously distributed, but supporting our third hypothesis.

Several large-scale studies have tried to identify environmental drivers of protistan diversity and community assembly [6,24,25,42,43,44]. However, the complexity of their diverse sizes, morphotypes, and diverse feeding modes complicates the detection of specific environmental drivers for a group of taxa. Species turnover (beta diversity) of Cryomonadida was high between large-scale contrasting habitats along a transect from blanket bog to a zonal forest. In particular, the strong gradient of soil acidity from bog to forest structured the Cryomonadida community. In contrast, gradients of physicochemical parameters of soil samples within a 10 × 10 m grassland site were rather low [6], but seasonal differences in plant biomass production and associated soil moisture were the main environmental factors structuring the small-scale community composition of Rhogostomidae. Although scientific reports of Rhogostomidae are scarce, the genus *Rhogostoma* was repeatedly found to be associated with plants, where it seems to feed on fungi, algae, and bacteria [10,11,45,46,47,48]. The affinity to plants also triggered the description of the first plant surface-associated *Rhogostoma* species, *R. epiphylla* [11]. Our phylogenetic analyses show an intermingling of soil and plant surface inhabiting *Rhogostoma* species and we further show a negative correlation of soil moisture and plant biomass to predict the Rhogostomidae community. Both of these findings may indicate that a diverse array of *Rhogostoma* species may be attracted to plant surfaces due to a certain degree of water availability in its vicinity. The organic theca of Rhogostomidae may protect the cells from water loss to a certain degree and may explain their increased dominance in drier conditions [6]. Additionally, the aperture of the theca of Rhogostomidae is particularly slim, potentially reducing water loss from the cell as an adaption to terrestrial life. Therefore, we expected clear differences in aperture depth between freshwater and terrestrial Rhogostomidae. However, we could not find clear evidence for this hypothesis since our phylogenetic analyses showed freshwater Rhogostomidae occasionally intermingling with terrestrial clades of Rhogostomidae. Considering their morphology (see Supplementary Materials for details), we further could not find a clear distinct difference of aperture depth or cell shape between freshwater and terrestrial Rhogostomidae. Instead, we found a negative correlation between aperture depth and the cell length/width ratio, which shows that the aperture depth is rather connected to overall cell size and shape and does not show direct adaption to environmental differences like water availability. Altogether this may indicate that *Rhogostoma* individuals independently of aperture depth and cell shape can tolerate drier conditions.

## 5. Conclusions

The Rhogostomidae are a particularly abundant taxon in terrestrial ecosystems and we show that they represent a surprisingly diverse family in the Cercozoa. Reliable species delimitation was possible by an integrative taxonomic approach. Clear patterns in beta diversity along environmental gradients indicate species turnover due to taxa specifically adapted to specific biotic and abiotic environmental conditions. However, specific morphological adaptions to microhabitat conditions could not be found in these character-poor and minute organisms. The invagination of the aperture and cell length to width ratio turned out to be the best morphological characters for species delimitation in Rhogostomidae.

## Figures and Tables

**Table 1:** Corresponding data of obtained *Rhogostoma* spp. strains.

Strain	CCAP Reference	Sampling Spots	Coordinates	Isolation Date	Habitat	Used Primers	Sequence Length (nt)
WM	1966/8	Germany, Rostock, Warnemünde	54.180267, 12.080450	December 2018	Sand beach, close to the Baltic Sea, algae-rich soil crust sample	EukA, 616F, 963R, EukB	1718
il-I	1966/13	Germany, Inden	50.875, 6.325	May 2019	Monoculture, bur clover (*Medicago sativa)*	EukA, 590F, 1300R, EukB	1627
IGS	1966/12	Germany, Inden	50.886, 6.317	May 2019	Monoculture, Barley shoot	EukA, 590F, 1300R, EukB	1767
3A	1966/11	Germany, Rostock, Warnemünde	54.180267, 12.080450	February 2019	Sand beach, close to the Baltic Sea, soil crust sample	EukA, cercomix, 963R, EukB	1619
B10	1966/6	Germany, Cologne	50.927186, 6.935997	December 2018	Courtyard of the Cologne Biocenter, leaf samples, phyllosphere	EukA, cercomix, 963R, EukB	1376
K8	1966/14	Germany, Cologne	51.025008, 6.751871	April 2019	Puddle with moss	EukA, cercomix, 963R, EukB	1606
K9	1966/15	Germany, Cologne	51.025041, 6.751496	April 2019	Litter from an Atlas cedar (*Cedrus atlantica*)	EukA, cercomix, 963R, EukB	1686
W2	1996/18	Austria, Vienna	48.200537, 16.370177	February 2019	Charles‘ Square, litter	EukA, 616F, 963R, EukB	1719
RC	1966/5	Germany, Cologne	50.927186, 6.935997	October 2017	Courtyard of the Cologne Biocenter, leaf samples, phyllosphere	EukA, cercomix, 963R, EukB	1720
1A	1966/9	Hilversum, Netherlands	52.247196, 5.167029	March 2019	Zanderij Crailoo, large pond, freshwater sample	EukA, cercomix, 963R, EukB	1708
WH4	1944/20	Germany, Wendershagen	50.899719, 7.735686	May 2019	Lichen from apple tree trunk	EukA, 590F, 1300R, EukB	1386
W3	1966/19	Austria, Vienna	48.207667, 16.366056	February 2019	Hofburg, lichen from cut branches	EukA, 616F, 963R, EukB	1721
1B	1966/10	Hilversum, Netherlands	52.247196, 5.167029	March 2019	Zanderij Crailoo, large pond, freshwater sample	EukA, cercomix, 963R, EukB	1719
TG4.2-II	1966/16	Germany, Cologne	50.920378, 7.105548	December 2018	Königsforst, forest litter	EukA, 590F, 1300R, EukB	909
TG4.2-IV	1966/17	Germany, Cologne	50.920378, 7.105548	December 2018	Königsforst, forest litter	EukA, cercomix, 963R, EukB	1721
B14	1966/7	Germany, Cologne	50.927186, 6.935997	December 2018	Courtyard of the Cologne Biocenter, leaf samples, phyllosphere	EukA, 590F, 1300R, EukB	1761

**Table 2:** Corresponding primers for SSU rDNA sequencing.

Used Primer	Primer Sequence
EukA [28]	general eukaryotic primer 5′-CCGAATTCGTCGACAACCTGGTTGATCCTGCCAGT-3′
EukB [28]	general eukaryotic primer 5′-CCCGGGATCCAAGCTTGATCCTTCTGCAGGTTCACCTAC-3′
590F [29]	general eukaryotic primer 5′-CGGTAATTCCAGCTCCAATAGC-3′
1300R [29]	general eukaryotic primer 5′-CACCAACTAAGAACGGCCATGC-3′
S963R_Cerco [5]	Cercozoa specific primer 5′- CAACTTTCGTTCTTGATTAAA-3′
S616F [5] *	Cercozoa specific primer 5′-TTAAAAAGCTCGTAGTTG-3′
S616F_Eocer [5] *	Cercozoa specific primer 5′-TTAAAAAGCGCGTAGTTG-3′

* Note: The primer “Cercomix” is a mixture of eight parts S616F and two parts S616F_Eocer.

## Supplementary Materials

The following are available online at https://www.mdpi.com/2076-2607/8/8/1123/s1, Figure S1: Morphological differences between the strains; visualized by boxplots (*n* = 20 for each strain). One-way ANOVA and post hoc Tukey test. Significant differences *p* < 0.05. Different species are highlighted by colour. Table S1: Morphological measurements of *Rhogostoma* strains. (means and standard deviation, *n* = 20 for each strain). Table S2: Genetical distance table for all molecularly known Rhogostomidae spp. and our isolated strains (in bold).

## Appendix A. Taxonomic Acts

***Rhogostoma kappa*** sp. nov. Öztoprak Figure 1J1–3, Type generating strain: 1A (March 2019, Crailoo, large pond; Hilversum, The Netherlands).

**Diagnosis**: 18S rDNA sequence, MN860275; Cell: spherical, laterally flattened, length: 16.5 ± 0.6 µm (10.5–23.1 µm), width: 11.21 ± 0.78 µm; length/width ratio about 1.5; shows clear zonation (apical to basal): (I) zone of nucleus, (II) food vacuoles, (III) aperture and multiple contractile vacuoles. Test: hyaline, smooth. Nucleus: at the apical end of the cell, spherical, about 5.7 ± 0.2 µm. Nucleolus: one, round; central to the nucleus. Aperture: basal, cleft-like, not flexible. Locomotion: actively creeping, filopodia lacked granules. No cysts observed. Freshwater. Prey: bacteria. Cell division: longitudinal, binary. **Etymology**: κ *kappa* [Greek] the tenth letter in Greek alphabet also referring to 河童 [kappa; Japanese] meaning “river child”; tortoise-like creature or imp in Japanese mythology inhabiting river streams and ponds referring to *Rhogostoma kappa*’s habitat.

***Rhogostoma florae*** sp. nov. Öztoprak. Figure 1G1–3 Type generating strain: K9 (April 2019, litter of *Cedrus atlantica*, Cologne, Germany). Other strains: K8. (Figure 1F1–3).

**Diagnosis**: 18S rDNA sequence, MN860283 (K9) and MN860284 (K8); Cell: oval but angular, length 11.7 ± 0.3 µm (8.8–11.7 µm), width: 8.6 ± 0.1 µm; length/width ratio about 1.3.; shows no clear zonation. Test: hyaline, smooth. Nucleus: spherical, about 4.2 ± 0.2 µm. Nucleolus: one, round; lateral to the nucleus. Aperture: basal, cleft-like, not flexible. Pseudopodia more often, but not restricted to form filopodia, branching and anastomosing. Locomotion: actively creeping, ceased to extract them as often later on in culture. No cysts observed. Plant litter. Prey: mainly bacteria, yeast observed as well. Cell division: longitudinal, binary. **Etymology:** *flora* [Latin] meaning flower, referring to the association between *Rhogostoma florae* and plant litter; also roman mythology *Flōra*—goddess of flowers and spring, in which this strain was isolated. Further, this species is dedicated to Flora Dumack in recognition of Dr. Dumack’s support even through the recent birth of his daughter Flora.

***Rhogostoma leviosa*** sp. nov. Öztoprak Figure 1H1–3 Type generating strain: W2 (February 2019, litter, Vienna, Austria).

**Diagnosis**: 18S rDNA sequence, MN860277; Cell: round, length: 9.9 ± 0.5 µm (7.7–11.8 µm), width: 8.2 ± 0.8 µm; length/width ratio about 1.2. Morphology as *R. florae* (–but significantly smaller). Test: hyaline, smooth. Nucleus: spherical, about 3.1 ± 0.2 µm. Nucleolus: one, round; central to the nucleus. No cysts observed. Floating stages observed. Granules evenly dispersed in the cell. Phyllosphere. Prey: mainly bacteria. Cell division: longitudinal, binary. **Etymology:** *leviosa* derived from *levo*- [Latin] meaning to rise, referring to the floating stages it formed earlier than other strains when starving. As a reference to the magic spell ‘Winggardium Leviosa’ in the Wizarding World, which is used to elevate objects.

***Rhogostoma radagasteri*** sp. nov. Öztoprak Figure 1O Type generating strain: TG4-IV (December 2018, Königsforst forest litter, Cologne, Germany). Other strains: TG4-II (Figure 1N1–3).

**Diagnosis**: 18S rDNA sequence, MN860281 (TG4.IV) and MN860282 (TG4-II); Cell: oval but angular, length: 11.7 ± 0.3 µm (8.5–14.2 µm), width: 10.1 ± 0.3 µm; length/width ratio about 1.2; shows clear zonation (apical to basal): (I) zone of nucleus, (II) food vacuoles, (III) aperture and multiple contractile vacuoles. Test: hyaline, smooth. Nucleus: spherical, about 4.6 ± 0.1 µm. Nucleolus: one, round; mostly central to the nucleus. Aperture: basal, cleft-like. Pseudopodia more often, but not restricted to, forming one-sided lamellipodia from which filopodia emerge. Locomotion: actively creeping, filopodia rarely extending to 40 µm. Soil. Prey: mainly bacteria. Cell division: longitudinal, binary. **Etymology:** *radagasteri* [Sindarin] derived from Radagast meaning ‘the tender of beasts’ of J.R.R. Tolkien’s Legendarium. Describing Radagast the brown, a wizard who lived in a forest, concerned about the well-being of the plant and animal world, referring to the isolation location which was from the Königsforst.

***Rhogostoma pseudocylindrica*** sp. nov. Öztoprak. Figure 1I1–3 Type generating strain: RC (October 2017, leaf sample (phyllosphere); Cologne, Germany).

**Diagnosis**: 18S rDNA sequence, MN860276; Cell: oval, slender length: 8.6 ± 1 µm (7.4–10.2 µm), width: 5.2 ± 1.7 µm; length/width ratio about 1.7. Test: hyaline, smooth. Nucleus: spherical, about 2.7 ± 0.3 µm. Nucleolus: one, round; central to the nucleus. Aperture: basal, cleft-like. Locomotion: actively creeping, filopodia. Observed to form floating stages. No cysts. Granules evenly dispersed in the cell body. Mostly two basally contractile vacuoles. Phyllosphere. Prey: mainly. Cell division: longitudinal, binary. **Etymology:** ψευδής *pseudḗs* [Greek] meaning false and lying referring to the morphological similarities of *R. cylindrica* which could lead to misidentification of both species.

***Rhogostoma kyoshii*** sp. nov. Öztoprak Figure 1A1–3 Type generating strain: WM (December 2018; soil crust, sand beach; Warnemünde, Germany).

**Diagnosis**: 18S rDNA sequence, MN860287; Cell: oval but angular, length: 12.4 ± 0.4 µm (10.3–15.2 µm), width: 10.7 ± 0.3 µm; length/width ratio about 1.2. Test: hyaline, smooth. Nucleus: spherical, about 3.9 ± 0.1 µm. Granules: observed to aggregate around the nucleus. Nucleolus: one, round; central to the nucleus. Aperture: basal, cleft-like; Locomotion: actively creeping. Filopodia. Soil crust. Prey: mainly bacteria and yeast. Cell division: longitudinal, binary. **Etymology:** *清* *kyoshi* [Japanese] meaning ‘pure’ referring to Avatar Kyoshi from the earth kingdom (“Avatar: The last Airbender” created by Michael Dante DiMartino and Bryan Konietzko). Referring to the habitat of *R. kyoshii* and the association with the dry surroundings of the earth kingdom.

***Rhogostoma medica*** sp. nov. Öztoprak Figure 1B1–3 Type generating strain: il-1 (May 2019; phyllosphere of bur clover; Inden, Germany).

**Diagnosis**: 18S rDNA sequence, MN860285; Cell: oval but angular, length: 14.8 ± 1.8 µm (10.1–17.5 µm), width: 13.3 ± 1.8 µm; length/width ratio about 1.2. Test: hyaline, smooth. Nucleus: spherical, about 4.7 ± 1.0 µm. Granules: evenly dispersed in the cell. Nucleolus: one, round; central to nucleus. Aperture: basal, cleft-like; Locomotion: actively creeping. Filopodia. Phyllosphere. Prey: mainly bacteria and yeast. Cell division: longitudinal, binary. **Etymology:** *medica* [Latin] meaning female doctor; also a kind of clover: alfalfa (*Medicago sativa*) referring to the origin and isolation habitat of this strain

***Rhogostoma tahiri*** sp. nov. Öztoprak Figure 1E1–3 Type generating strain: Biozentrum10 (December 2018, phyllosphere, Cologne, Germany).

**Diagnosis**: 18S rDNA sequence, MN860279; Cell: oval, length: 10.9 ± 0.3 µm (8.7–12.6 µm), width: 9.9 ± 0.4 µm; length/width ratio about 1.2; shows no clear zonation. Test: hyaline, smooth. Nucleus: spherical, about 5.8 ± 0.1 µm. Nucleolus: one, round; mostly central to the nucleus. Aperture: basal, cleft-like. Locomotion: actively creeping. Filopodia. Soil crust. Prey: mainly bacteria; also *S. cerevisiae*. Cell division: longitudinal, binary. **Etymology:** طاهر *Tahir* [Arabic] meaning sparkling, clean. This species is dedicated to my nephew Tahir Said, who was born while I conducted this study and brought me rays of joy.

***Rhogostoma bowseri*** sp. nov. Öztoprak Figure 1P1–3 Type generating strain: Biozentrum14 (December 2018; phyllosphere, Cologne, Germany).

**Diagnosis**: 18S rDNA sequence, MN860289; Cell: roundish, length: 18.4 ± 0.4 µm (14.6–20.8 µm), width 16.6 ± 0.4 µm; length/width ratio about 1.1. Test: hyaline, bears xenosomes, often associated with debris and bacteria. Nucleus: spherical, lateral in cell, about 6.6 ± 0.1 µm. Nucleolus: one, round; central to nucleus. Aperture: cleft-like. Under culture conditions no xenosomes. Filopodia or lamellipodia. No cysts. Granules dispersed laterally in the cell body. Locomotion: actively creeping. Phyllosphere. Prey: mainly bacteria. Cell division: longitudinal, binary. **Etymology:** Bowser is the great antagonist of the Mario franchise. This species is dedicated to him due to the morphological resemble of *R. bowseri*’s xenosomes and its similarities to Bowser’s turtle shell.

***Rhogostoma karsteni*** sp. nov. Öztoprak Figure 1D1–3 Type generating strain: 3A (Februrary 2019; soil crust, Rostock, Germany).

**Diagnosis**: 18S rDNA sequence, MN860288; Cell: oval, length: 14.7 ± 2.1 µm (11.5–18.7 µm), width 12.6 ± 2.1 µm; length/width ratio about 1.1.; shows no clear zonation. Test: hyaline, smooth. Nucleus: spherical, lateral in the cell, heterogenous structure, about 5.8 ± 1 µm. Nucleolus: one, round; lateral to the nucleus. Aperture: cleft-like, protuberated. Filopodia or lamellipodia. No cysts. Granules dispersed laterally in the cell body. Locomotion: actively creeping. Soil crust. Prey: mainly bacteria. Cell division: longitudinal, binary. **Etymology:** This species is dedicated to Prof. Dr. Ulf Karsten.

***Rhogostoma absidea*** sp. nov. Öztoprak Figure 1L1–3 Type generating strain: W3 (February 2019; Lichen from fresh-cut tree branches, Vienna, Austria).

**Diagnosis**: 18S rDNA sequence, MN860278; Cell: round, apical—basal compressed, length: 8.1 ± 0.9 µm (6.2–9.6 µm), width: 8.7 ± 0.4; length/width ratio about 1.1. Test: hyaline, smooth Nucleus: spherical, about 3 ± 0.2 µm. Nucleolus: one, round; central to the nucleus. Aperture: basal, cleft-like, protuberated. Locomotion: actively creeping. Filopodia. Observed to form floating stages. No cysts. Granules evenly dispersed in the cell body. Phyllosphere. Prey: mainly bacteria. Cell division: longitudinal, binary. **Etymology:** *abs* [Latin] meaning without, no; *idea* [Latin] meaning idea. Referring to my state of mind after describing and naming various species and lacking inspiration for a species name for this last one.
