# Supplementary material for: What Drives the Diversity of the Most Abundant Terrestrial Cercozoan Family (Rhogostomidae, Cercozoa, Rhizaria)?
Source: Microorganisms. 2020 Jul 26;8(8):1123. doi: 10.3390/microorganisms8081123 (PMC7463998; doi:10.3390/microorganisms8081123)
Supplement: Supplementary file 1 [file microorganisms-08-01123-s001.pdf]

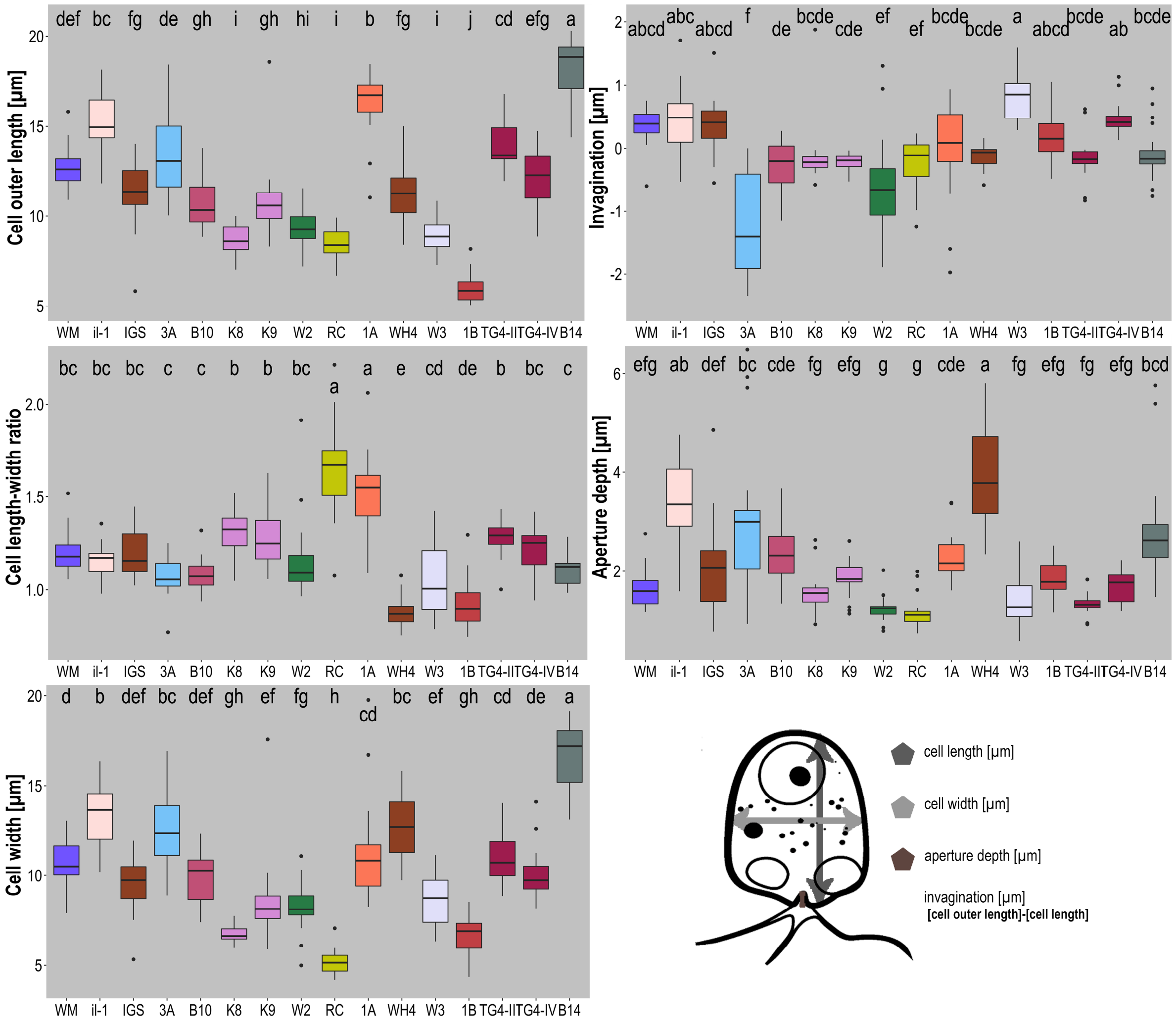

Supplementary Figure 1: Morphological differences between the strains; visualized by boxplots (N = 20 for each strain). One-way ANOVA and post hoc Tukey test. Significant differences p<0.05. Different species are highlighted by colour.

Supplementary Table 1: Morphological measurements of *Rhogostoma* strains. (means and standard deviation, N = 20 for each strain).

	Cell middle length [μm]	Cell outer length [μm]	Aperture invagination [μm]	Cell width [μm]	Cell length to width Ratio	Nucleus length [μm]	Nucleus width [μm]	Nucleus length to width ratio	Nucleolus length [μm]	Nucleolus width [μm]	Nucleolus length to width ratio	Nucleus to nucleolus ratio	Aperture depth
WM	12.41±1.282	12.77±1.2	0.37±0.29	10.7±1.35	1.2±0.12	3.89±0.44	3.76±0.36	1.04	1.01±0.21	0.95±0.2	1.08	4.01	1.64±0.41
il-1	14.75±1.75	15.19±1.69	0.44±0.52	13.26±1.82	1.15±0.1	5.03±1.12	4.7±0.96	1.07	1.71±0.33	1.68±0.29	1.03	2.99	3.38±0.84
IGS	10.94±1.88	11.3±1.84	0.36±0.45	9.51±1.5	1.19±0.12	3.86±1.01	3.51±0.99	1.11	1.51±0.36	1.51±0.38	1.02	2.6	2.08±0.96
3A	14.68±2.07	13.47±2.43	-1.23±0.77	12.6±2.07	1.07±0.11	5.78±1.01	5.49±0.94	1.05	1.77±0.31	1.73±0.39	1.03	3.33	3.05±1.49
B10	10.86±1.54	10.6±1.37	-0.27±0.4	9.92±1.48	1.08±0.09	3.72±0.18	3.3±0.41	1.15	1.38±0.39	1.28±0.31	1.08	2.84	2.38±0.64
K8	8.86±0.85	8.72±0.88	-0.14±0.49	6.66±0.46	1.31±0.12	3.46±0.51	3.32±0.49	1.05	1.17±0.25	1.08±0.21	1.08	3.13	1.55±0.42
K9	11.07±2.09	10.85±2.09	-0.22±0.14	8.57±2.33	1.29±0.16	4.3±0.65	3.94±0.64	1.11	1.57±0.37	1.46±0.32	1.08	2.83	1.85±0.37
W2	9.91±1.01	9.3±1.07	-0.61±0.8	8.22±1.34	1.16±0.22	3.09±0.53	2.94±0.49	1.05	1.24±0.29	1.11±0.24	1.12	2.59	1.25±0.26
RC	8.65±0.88	8.47±0.81	-0.61±1.67	5.19±0.68	1.65±0.25	2.68±0.4	2.31±0.36	1.17	0.76±0.16	0.77±0.19	1.01	3.63	1.18±0.32
1A	16.56±2.57	16.56±2.13	-0.02±0.77	11.23±2.84	1.51±0.24	5.74±1.15	5.3±1.23	1.09	1.24±0.18	1.1±0.31	1.59	4.72	2.34±0.51
WH4	11.3±1.74	11.17±1.69	-0.13±0.18	12.71±1.81	0.88±0.08	4.32±0.58	3.89±0.8	1.14	1.46±0.27	1.37±0.25	1.07	3.05	3.85±0.94
W3	8.07±0.91	8.9±0.86	0.83±0.39	8.68±1.52	1.05±0.2	2.99±0.87	2.73±0.94	1.17	1.35±0.45	1.26±0.45	1.09	2.39	1.44±0.58
1B	5.84±0.742	6.0±0.83	0.15±0.38	6.74±1.015	0.9±0.15	2.69±0.51	2.37±0.52	1.15	0.91±0.27	0.84±0.28	1.1	3.15	1.86±0.39
TG4-II	14.1±1.57	13.95±1.49	-0.16±0.34	11.0±1.37	1.28±0.1	4.47±0.42	3.93±0.45	1.15	1.77±0.28	1.68±0.25	1.05	2.6	1.33±0.22
TG4-IV	11.66±1.39	12.12±1.49	0.47±0.24	10.03±1.42	1.22±0.12	4.49±0.7	3.97±0.7	1.14	1.93±0.4	1.8±0.4	1.08	2.44	1.69±0.33
B14	18.37±1.55	18.29±1.49	-0.08±0.43	16.61±1.7	1.11±0.08	6.62±0.74	6.4±0.78	1.03	2.41±0.38	2.38±0.48	1.02	2.82	2.82±1.08

Table 2: Genetical distance table for all molecularly known Rhogostomidae spp. and our isolated strains (in bold).

			1	**2**	3	**4**	5	**6**	**7**	**8**	**9**	10	**11**	**12**	**13**	**14**	15	**16**	**17**	**18**	**19**	**20**	**21**	22	23	24	25	26
1		*Sacciforma sacciformis*	-																									
**2**	**1A**	***R. kappa***	0.084	-																								
3		*R. cylindrica*	0.07	0.054	-																							
**4**	**W3**	***R. absidea***	0.082	0.059	0.014	-																						
5		*R. schuessleri*	0.085	0.057	0.012	0.016	-																					
**6**	**B10**	***R. tahiri***	0.075	0.054	0.017	0.019	0.014	-																				
**7**	**IGS**	***R. epiphylla***	0.090	0.063	0.02	0.023	0.026	0.018	-																			
**8**	**WM**	***R. kyoshii***	0.093	0.075	0.037	0.039	0.036	0.029	0.023	-																		
**9**	**il-1**	***R. medica***	0.083	0.059	0.028	0.028	0.029	0.022	0.012	0.027	-																	
10		*R. epiphylla*	0.074	0.065	0.021	0.02	0.021	0.018	0.004	0.024	0.01	-																
**11**	**WH4**	***R. epiphylla***	0.067	0.049	0.023	0.021	0.022	0.02	0.005	0.029	0.01	0	-															
**12**	**RC**	***R. pseudo-cylindrica***	0.08	0.058	0.01	0.015	0.016	0.017	0.022	0.037	0.028	0.022	0.021	-														
**13**	**W2**	***R. leviosa***	0.078	0.061	0.011	0.017	0.016	0.014	0.025	0.039	0.026	0.02	0.02	0.016	-													
**14**	**K8**	***R. florae***	0.068	0.062	0.022	0.024	0.023	0.024	0.029	0.041	0.031	0.028	0.026	0.024	0.012	-												
15		‚*Capsellina* sp.'	0.082	0.07	0.027	0.03	0.031	0.029	0.042	0.048	0.035	0.032	0.031	0.03	0.02	0.004	-											
**16**	**K9**	***R. florae***	0.074	0.067	0.022	0.026	0.028	0.023	0.032	0.044	0.031	0.027	0.025	0.026	0.015	0	0.004	-										
**17**	**B14**	***R. bowseri***	0.084	0.062	0.013	0.023	0.019	0.019	0.029	0.047	0.032	0.027	0.03	0.022	0.021	0.029	0.037	0.033	-									
**18**	**3A**	***R. karsteni***	0.086	0.058	0.007	0.02	0.016	0.012	0.024	0.042	0.026	0.02	0.022	0.017	0.016	0.024	0.034	0.028	0.012	-								
**19**	**TG4-IV**	***R. radagasteri***	0.084	0.059	0.018	0.02	0.022	0.021	0.025	0.041	0.034	0.026	0.026	0.023	0.023	0.029	0.035	0.031	0.025	0.022	-							
**20**	**TG4-II**	***R. radagasteri***	0.075	0.059	0.025	0.03	0.03	0.028	0.033	0.049	0.038	0.037	0.035	0.035	0.031	0.038	0.047	0.038	0.028	0.024	0.005	-						

**21**	**1B**	***R. micra***	0.081	0.06	0.034	0.034	0.036	0.032	0.042	0.049	0.044	0.042	0.037	0.037	0.033	0.041	0.045	0.04	0.035	0.033	0.029	0.037	-					
22		*R. micra*	0.088	0.07	0.034	0.037	0.039	0.033	0.042	0.049	0.046	0.042	0.041	0.038	0.036	0.039	0.046	0.04	0.04	0.036	0.031	0.038	0.009	-				
23		*R. minus*	0.087	0.075	0.033	0.039	0.038	0.033	0.047	0.048	0.044	0.04	0.038	0.039	0.036	0.037	0.041	0.037	0.039	0.041	0.033	0.045	0.012	0.01	-			
24		*Rhogostoma* sp.	0.085	0.076	0.039	0.039	0.038	0.033	0.045	0.057	0.048	0.045	0.045	0.036	0.032	0.036	0.043	0.035	0.039	0.04	0.035	0.049	0.012	0.011	0	-		
25		*R. minus*	0.091	0.08	0.035	0.04	0.039	0.035	0.049	0.05	0.047	0.042	0.04	0.04	0.037	0.038	0.043	0.039	0.041	0.041	0.035	0.046	0.013	0.01	0.001	0	-	
26		*R. minus ,Lecythium* sp.'	0.098	0.089	0.043	0.049	0.047	0.046	0.058	0.058	0.056	0.05	0.052	0.049	0.046	0.047	0.052	0.047	0.05	0.05	0.043	0.061	0.022	0.018	0.009	0.009	0.008	-
